# Sexual dysfunction associated with prostate cancer treatment in Japanese men: a qualitative research

**DOI:** 10.1007/s00520-021-06728-2

**Published:** 2022-01-01

**Authors:** Saeko Hayashi, Fumiko Oishi, Kazuki Sato, Hiromi Fukuda, Shoko Ando

**Affiliations:** 1 Department of Nursing, Doctoral Course, Graduate School of Medicine, Nagoya University, Nagoya, Japan; 2 College of Nursing, Aichi Medical University, 1-1, Yazakokarimata, Nagakute-city, Aichi Pref. 480-1195 Japan; 3 School of Nursing, Seirei Christopher University, Shizuoka, Japan; 4 Department of Integrated Health Sciences, Nursing for Advanced Practice, Graduate School of Medicine, Nagoya University, Nagoya, Japan; 5 Department of School Health Sciences, Aichi University of Education, Aichi, Japan

**Keywords:** Sexual dysfunction, Prostate cancer treatment, Japanese men, Qualitative analysis

## Abstract

**Purpose:**

We investigated the experiences of Japanese men with sexual dysfunction associated with various prostate cancer treatments.

**Methods:**

We included 38 Japanese men who underwent the following initial treatments for prostate cancer: radical prostatectomy (*n* = 10), external beam radiotherapy (*n* = 12), brachytherapy (*n* = 5), and androgen deprivation therapy (*n* = 11). Semi-structured interviews were conducted regarding sexual dysfunction associated with prostate cancer treatment. Data were analyzed using a content analysis method. To obtain a unique experience for each treatment, we confirmed and organized the treatment method from which the code that constituted each category was derived. The category reliability was calculated based on Scott’s formula for the matching rate of the classification by three qualitative researchers. The criterion for good reliability was set at 70%.

**Results:**

Japanese men with sexual dysfunction associated with prostate cancer treatments experienced the following: a desire to maintain sexual function and conflict in decision-making concerning the initial treatment for prostate cancer; a loss of values related to sexual dysfunction; an uncertainty regarding the consequences of sexual dysfunction; a sense of calm with fewer adverse effects of sexual dysfunction at the early treatment stage; an effort to accept sexual dysfunction; and management of their changed body at the later treatment stages. The concordance rates for the categories were 70% and 78%. Additionally, there were glimpses of experiences common to all treatments and trends in treatment-specific experiences.

**Conclusion:**

It is necessary to provide care based on the experience of Japanese men with sexual dysfunction after prostate cancer treatment.

## Background

Prostate cancer is the most common type of cancer among men in Japan and worldwide [1, 2]. The main treatment options for prostate cancer are radical prostatectomy (prostatectomy), external beam radiotherapy (EBRT), and brachytherapy (LDR). Japan’s Prostate Cancer Practice Guidelines [3] state that androgen deprivation therapy (ADT) has a promising therapeutic effect; thus, after careful discussions with physicians, many patients choose to undergo ADT monotherapy, considering the clinical stage and risk classification of the tumor, their age, health, and possible complications. However, prostate cancer treatment generally causes deteriorated sexual function in men [4–6]. Treatment-related sexual dysfunction affects men’s quality of life and is associated with mental distress, depression, and changes in lifestyle and relationships [7–10]. Therefore, other countries use drug medications and erectile aids for sexual dysfunction, and perform psychological interventions for patients and their partners to maintain healthy sexual relationships [11].

Compared to American men, Japanese men with prostate cancer have a more pronounced decline in libido, erectile function, and the ability to achieve orgasm; nevertheless, they have fewer complaints of sexual trouble [12]. Therefore, ethnicity must be considered while treating Japanese men with prostate cancer, as they are not openly anxious regarding their sexual issues; sexual dysfunction is considered a non-issue in Japan [13, 14].

In Japan, previous studies involving men with sexual dysfunction because of prostate cancer treatment were limited by their small sample sizes and unspecified treatments. Additionally, although libido, erection, ejaculation, and orgasm disorders have unique anatomical and physiological characteristics, they are all considered “sexual dysfunctions.” These factors have obscured the details of sexual experiences and hindered the establishment of a support system [14]. Thus, we examined the experiences of Japanese men with sexual dysfunction associated with various prostate cancer treatments. Our findings will help in the development of effective management strategies for sexual dysfunction associated with prostate cancer, and may also encourage further quantitative studies, which would help highlight patient experiences related to sexual dysfunction or help evaluate the relevant interventions.

## Methods

### Study design and participants

Men’s experiences with sexual dysfunction associated with prostate cancer treatment are guided by the complex and diverse backgrounds of individual men. We performed a qualitative inductive study, which incorporates the natural everyday context as it exists in its complex form, uncovers new aspects of the phenomenon, and is effective for various and complicated problems [15]. As this study focused on Japanese people who are generally reluctant to express sexual concerns, we were apprehensive that the participant number could decrease if the criteria were too detailed, as in other studies on sexuality in Japan. Considering that this was a qualitative survey, we aimed to secure an adequate number of participants to reach data saturation. The inclusion criteria were as follows: patients who selected prostatectomy, EBRT, LDR, or ADT monotherapy as the initial prostate cancer treatment and those who provided written informed consent for study participation. The exclusion criteria were as follows: patients with physical or mental difficulties, who were challenging to examine, and patients aged < 20 years.

Participants were recruited through the Prostate Cancer Patients’ Association mailing list, which includes members throughout Japan. Additionally, six hospitals and clinics that provide prostate cancer treatment were selected for convenience, and a research cooperation request form was created. The participants were recruited at the outpatient department. Age, marital status, parenting experience, employment at the time of initial treatment, and medical history were considered, to obtain diversity in the experiences of sexual dysfunction associated with prostate cancer treatment.

### Procedure

Participants were requested to provide their background information, sexual dysfunction experienced, and frequency of sexual intercourse. One-on-one semi-structured interviews were conducted by a mature female researcher, who is trained in sex counseling, to obtain information regarding men’s sexual dysfunction. The interviews were conducted in Japanese, using an expert and literature-based interview guide (Table 1), to obtain detailed, comprehensive, and unrestrained narratives. Data were collected from February 2019 to November 2020. All audio and verbatim recordings were stored in a password-protected computer. This study was conducted in accordance with the Standards for Reporting Qualitative Research guidelines [16].

**Table 1:** Interview guide

Please briefly explain the process leading up to the diagnosis of prostate cancer
Please tell me about the process leading up to the first treatment decision, including episodes
Please tell me about the first time you felt a change in sexual function after treatment, including the episode
Are there any changes, for example, in body structure or function, awareness of yourself, or relationships due to changes in sexual function? If so, please elaborate
What was the most painful change in sexual function? Please elaborate

### Data analysis

Using the content analysis method of Berelson [17], categories were formed according to similarity of meaning, content, and category names that accurately represented the similarity. The frequency of appearance of the recording units included in each category was quantified and tabulated for each category. Finally, to obtain a unique experience for each treatment, we confirmed and organized the code constituting the category from which the treatment method was derived.

The process of analysis was repeated among qualitative researchers specializing in cancer nursing to ensure certainty. Category reliability was calculated based on Scott’s formula for the matching rate of classifications by three qualitative researchers. The criterion for good reliability was set at 70%.

## Results

**Fig. 1:**
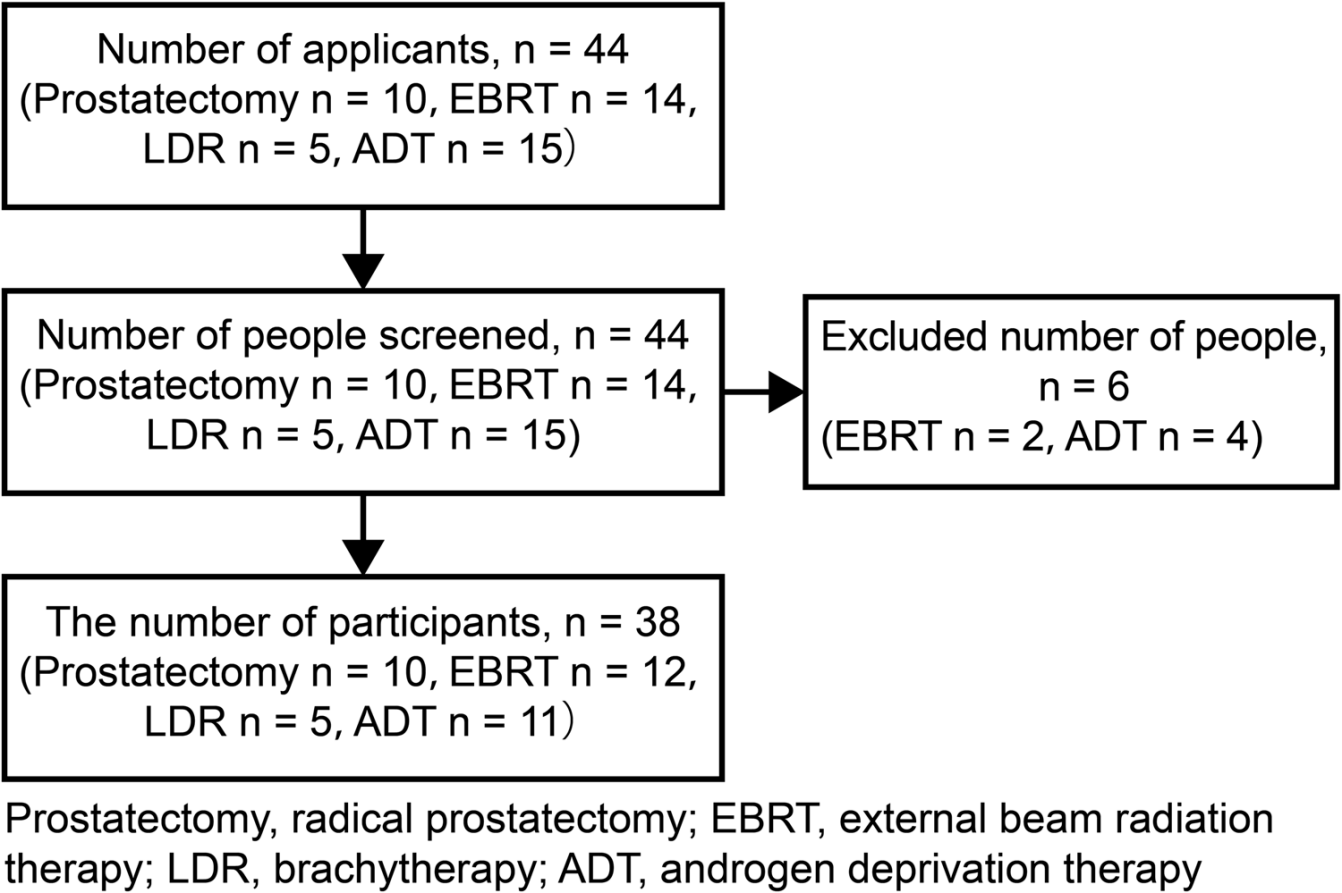
Participant screening flow chart

The participants were examined, as described in the flow chart (Fig. 1). There were 10, 12, 5, and 11 cases of prostatectomy, EBRT, LDR, and ADT, respectively (Table 2). Changes in sexual function peculiar to treatment were observed (Table 3). We found that patients who underwent EBRT and LDR maintained the frequency of sexual intercourse even after treatment (Table 4).

**Table 2:** Demographics of participants (*n* = 38)

Initial treatment		Prostatectomy (*n* = 10)	EBRT (*n* = 12)	LDR (*n* = 5)	ADT (*n* = 11)
Age at the start of primary intervention	Median (range)	63 (55–69)	61.5 (47–73)	63 (50–70)	75 (69–82)
Age at the time of survey	Median (range)	69.5 (57–79)	62 (50–75)	65 (53–74)	80 (72–84)
Years since primary intervention	Median (range)	6.5 (2–10)	6 (1–10)	2 (1–4)	3 (0–11)
Marital status	Married	10	11	3	9
	Divorced			1	1
	Single		1	1	1
Parenting experience	Yes	7	10	4	11
	No	3	2	1	
Job at the start of primary intervention	Business owner	1	3	1	
	Employee	6	7	4	3
	Farmer	1			
	Part-time job	2	1		1
	Retired		1		7
Medical history	Diabetes	0	2	0	0
	High blood pressure	4	3	2	8
	Heart disease	0	4	0	0
	Chronic kidney disease	0	1	0	1

*Prostatectomy*, radical prostatectomy; *EBRT*, external beam radiotherapy; *ADT*, androgen deprivation therapy

**Table 3:** Changes in sexual function experienced by participants

Initial treatment		Prostatectomy (*n* = 10)	EBRT (*n* = 12)	LDR (*n* = 5)	ADT (*n* = 11)
Changes in sexual function experienced (participant description and number of participants described)	Decreased libido				9
	None or poor erection	10	6	2	8
	None or decrease in semen	10	8	5	7
	Changes in semen properties (bloody, thick, colorless, and transparent, smell changed)		8	1	
	Tender orgasm	4	3	5	
	Ejaculatory pain or discomfort			2	

*Prostatectomy*, radical prostatectomy; *EBRT*, external beam radiotherapy; *ADT*, androgen deprivation therapy; *LDR*, brachytherapy

**Table 4:** Frequency of sexual intercourse at the start of initial treatment and at the time of the investigation

Initial treatment	Prostatectomy (*n* = 10)		EBRT (*n* = 12)		LDR (*n* = 5)		ADT (*n* = 11)	
	At the start of primary intervention	At the time of survey	At the start of primary intervention	At the time of survey	At the start of primary intervention	At the time of survey	At the start of primary intervention	At the time of survey
More than once/month	4		5	2	5	3		
Sometimes	1			1			4	1
None	5	10	7	9		2	7	10

*Prostatectomy*, radical prostatectomy; *EBRT*, external beam radiotherapy; *ADT*, androgen deprivation therapy; *LDR*, brachytherapy

The interviews lasted 27–100 min (mean: 51.9 min; standard deviation, 17.6). Patients were analyzed based on the procedure they underwent, and 547 codes were integrated into 54, 17, and 6 subcategories, categories, and core categories, respectively (Table 5). The core categories are presented below. The descriptions in parentheses indicate the selected treatment method.

**Table 5:** Experiences of men with sexual dysfunction associated with prostate cancer treatment

Core category (6)	Category (17)	Appearance frequency of category	Category structure with each treatment				Subcategory (54)
			Prostatectomy	EBRT	LDR	ADT	
Desire and conflict to maintain sexual function in decision-making concerning the initial treatment for prostate cancer	Desire to maintain sexual function and search for treatment methods that can maintain sexual function	5%	●	●	●		Have a strong desire to maintain sexual function
							Search for treatment methods with less effect on sexual function and find physicians or hospitals that provide the desired treatment
							Select a treatment method that can maintain sexual function
	Disagreement with family members who only focus on cancer cure in treatment strategy	2%	●	●	●		Face disagreement with family members who only focus on cancer removal, not sexual function, concerning the selection of a treatment strategy
							Avoid discussing treatment options with wife who only focuses on cancer removal when selecting treatment method
	Conflict to give up sexual function for cancer cure	7%	●	●		●	Face fear of losing sexual function and sex life before treatment
							Feel resigned to preserving sexual function because of fear of recurrence or metastasis
							Give up sexual function to leave treatment options in case of recurrence
							Accept the possibility of losing sexual function and sex life by considering unused functions unnecessary
	Grief of losing sexual function and sex life and discussion regarding a relationship that replaces sex life	1%				●	Face fear of losing sexual function and sex life after treatment and experiences grief
							Consider alternatives that replace sex life with partner in preparation for the loss of sexual function and sex life
Loss of values related to sexual dysfunction	Loss of confidence and agony as a man	7%	●	●	●	●	Lose confidence as a man because of erectile dysfunction
							Lose confidence as a man because of ejaculatory dysfunction
							Feel lonely that the body does not react sexually to attractive individuals
							Struggle to accept oneself as a man
	Change and agony in intimate relationship with partner	13%	●	●	●	●	Marital relationship weakens because of lack of understanding from wife regarding the distress associated with sexual dysfunction
							Relationship ends because partner cannot accept relationship without sex
							Suffer from giving up sex life as a married couple
							Feel sorry for wife concerning loss of sex life
							Marital relationship worsens and motivation to support family diminishes
	Fear of losing envisioned happiness because of loss of sexual function and reproductive capability	3%		●	●		Worried concerning future relationships and marriage in the event of loss of sexual function or reproductive capability
							Fear of losing reproductive capability and the joy of becoming a parent
							Discontinue treatment to maintain hope for having children
	Regret of past choices because of unexpected losses	4%	●				Regret past choices due to unexpected losses
Uncertainty concerning the consequences of sexual dysfunction	Repetition of recovery-related cycle of expectation and disappointment	12%	●	●	●		Realize the onset of sexual dysfunction and feels disappointed
							Feel frustrated because of the inability to satisfy sexual desire
							Aware of the onset of sexual dysfunction and expects recovery
							Repeat expectation and disappointment toward the recovery of sexual function
	Relief by recovery of sexual function /understanding of the recoverability of sexual function in the process of the repetition	4%		●	●		Understand that sexual function will not be recovered in the process of the repetition
							Feel relieved to be able to have sex life because of preservation/recovery of erectile function
							Feel relieved to be able to maintain sexual function and sex life
Sense of calm with less adverse effects of sexual dysfunction	Maintained peace of mind due to reduced sexual desire/sex life before treatment	8%	●	●		●	There is little change after treatment because of reduced sexual desire/sex life before treatment
	Relief because of the ability to control sexual emotions	1%				●	Feel relieved because of the ability to control sexual emotions
Effort to accept sexual dysfunction	Search for sympathy and shift to humor about sexual dysfunction	5%		●	●	●	Confess sexual dysfunction to the wife and gain sympathy and acceptance
							Confess sexual dysfunction to friends of the same generation, gain sympathy, and turn into laughter
	Enjoyment of daily life regardless of sexual dysfunction	7%	●	●		●	Enjoy hobbies regardless of sexual dysfunction
							Focus on work regardless of sexual dysfunction
							Engage in volunteer work regardless of sexual dysfunction
							Drink alcohol and forget about the situation when feeling distress about sexual dysfunction
	Reconsideration of life and personal values	14%	●	●	●	●	Realize that there are several remaining values other than sexual function
							Realize that sexual dysfunction does not damage previous hard work or marital love
							Perceive that sexual dysfunction occurs in everyone with aging
							Realize the effect of treatment
							Be satisfied with family life cycle and accept sexual dysfunction
							Considering that the degree of sexual dysfunction with this treatment is better than that with other treatment
							Considering that remaining alive is more important than having a sexual function
							Considering that dysuria is a bigger problem than sexual dysfunction
							Feel relieved by presuming that the wife also does not need sex life
Management of changed body	Search for methods to control sexual emotions and orgasms without relying on professionals	4%	●				Avoid stimulation to prevent sexual arousal
							Control sexual arousal
							Seek other methods to achieve orgasm
	Seek professional support to solve problems regarding sexual dysfunction	3%	●	●			Consult with the physician about concerns regarding sexual dysfunction
							Discuss treatment options for sexual dysfunction with physician

*Prostatectomy*, radical prostatectomy; *EBRT*, external beam radiation therapy; *LDR*, brachytherapy; *ADT*, androgen deprivation therapy

### Desire and conflict to maintain sexual function in decision-making concerning the initial treatment for prostate cancer

Men were eager to maintain sexual function, but faced the possibility of sexual function impairment after treatment. They required a treatment that would have minimal effect on sexual function. Their spouses and other family members could not understand their feelings toward maintaining sexual function. Therefore, the men avoided consulting them and sought treatment individually. Before treatment initiation, some patients gave up sexual function, considering a possibly heightened “risk of future recurrence/metastasis” if they were too obsessed with the desire to maintain sexual function. The men and their partners searched for alternatives to sexual relationship because of the threat of sexual function loss post-treatment.

“The first hospital recommended complete removal. However, I checked the effect on sexual function and searched for an institution that could perform SBRT and spacers.” (EBRT)

“I avoided consulting with my wife who misunderstood that complete removal would be better because it would wipe out cancer.” (LDR)

“I was worried concerning future recurrence; therefore, I gave up on sexual function and chose total resection instead of nerve-sparing prostatectomy.” (Prostatectomy)

“My partner and I talked that even if we could not have sex after the treatment, we might share the fun and discomfort that would happen in everyday life and continue physical contact without insertion.” (ADT)

### Loss of values related to sexual dysfunction

Men presented loss of confidence and changes in their intimate relationship with their partners. The primary cause of these changes was the women’s perceived inability to understand the men’s pain from sexual dysfunction, leading to a diminished marital relationship. Another cause of the changes in their intimate relationship was the loss of intercourse. These losses undermined the men’s motivation to play their roles. Moreover, lost reproductive function was perceived as a threat to the building of intimate relationships and the joy of becoming a parent. Men struggled to maintain their core values, regretted past choices, and were overwhelmed by unexpected losses.

“My wife and I still have the energy to live. We feel disappointed to live after prostatectomy without having a sexual life.” (Prostatectomy)

“My wife is insensitive to sexual dysfunction. I feel that she does not understand my emotions. My feeling toward her has stopped. After developing erectile dysfunction, our marital relationship has become lackluster.” (Prostatectomy)

“Someone advised me to remarry a specific woman. However, this woman told me that she did not like a man who could not have an erection. Therefore, I gave up on remarriage.” (ADT)

“Like the sun and the moon, I can work hard only if I have a sexual life. I lost my sexual life and motivation for work.” (Prostatectomy)

“I discontinued the treatment without consulting my physician and stored my sperm because I wanted to have a partner in the future and to retain the possibility of happiness of having my child. However, the cancer metastasized to the bones, causing pain. I should be aware of my life expectancy.” (EBRT)

“The physicians said they could maintain the erectile nerves. At that time, I was afraid of cancer metastasis. Therefore, I thought that it would be better to remove it completely. I did not expect that my sexual or marital life would change. Maybe I should have saved the erectile nerves.” (Prostatectomy)

### Uncertainty concerning the consequences of sexual dysfunction

The men hoped to maintain sexual function and resume their sexual life. Some were relieved to find that their sexual function was unaffected by the treatment. Others believed and expected recovery from sexual dysfunction. The cycle of recovery-related expectation and disappointment was repetitive. The repetition led some men to understand that sexual function would not return.

“I have retained erectile function, but not semen production. I usually feel very upbeat when the semen passes through the urethra. However, after treatment, that sensation has vanished. I expect a climax, although it ends in a pipe dream. It is my eternal hope to get back the sexual ecstasy.” (LDR)

“I found that the semen had turned like soup stock. It made me look like a fool who had expected the recovery of erectile function. I gave up, thinking that the semen dried up because of my old age and the treatment.” (EBRT)

### Sense of calm with less adverse effects of sexual dysfunction

Some men had low libido and a declining sexual life before the treatment. Thus, they did not consider that their sexual life had changed after sexual dysfunction occurred. Additionally, the sexual dysfunction enabled them to suppress sexual impulses, bringing a sense of relief.

“Although I lost sexual function, we had been sexless earlier. Therefore, it did not affect our family life.” (Prostatectomy)

“I am relieved that I have no erections, ejaculations, and desire to have an intercourse. I feel that I have been able to get out of the woods and reach a state of enlightenment. I have been suffering from conflicts between emotions and reasons. However, ADT drove out the conflict quickly.” (ADT)

### Effort to accept sexual dysfunction

Men shared their concerns regarding sexual dysfunction with their wives and friends, who empathized with them and helped them turn their worries into humor. They also enjoyed what they could do without being anxious about their sexual dysfunction. Moreover, the men re-evaluated their values, with consideration of the effect of the treatment, their family histories, and their health.

“When I told my wife that radiotherapy had damaged my sexual function, she said, “I do not want to show my surgical wounds, therefore it is all right.”” (EBRT)

“When I talked about my erectile dysfunction, a friend of my generation said, “I am in the same situation.” He talked about a woman who was joking and laughing regarding erectile disorder.” (ADT)

“I enjoy growing vegetables. Thus, I have no time to think of sexual dysfunction.” (Prostatectomy)

“I am satisfied to think that I had fully experienced the sexual bond between a husband and a wife.” (ADT)

### Management of changed bodies

Men were trying to manage their altered bodies by exploring how to deal with sexual emotions and achieve orgasm without resorting to specialists. Moreover, discussion with a physician concerning treatment for sexual dysfunction was a powerful support for regaining sexual life even with a changed body.

“I learned what behavior would satisfy me. Therefore, I do it occasionally when I am frustrated.” (Prostatectomy)

“I can tell my physician frankly that my sexual function is declining. It is encouraging because my physician cares about prescribing an erectile medicine.” (EBRT)

The concordance rates of the classification to the categories calculated were 70.0% (95% confidence interval [59.4–80.6]) and 78.0% (95% confidence interval [67.2–88.2]), respectively.

## Discussion

### Desire and conflict to maintain sexual function in decision-making concerning the initial treatment for prostate cancer

When selecting treatment method, the men were insistent about maintaining their sexual function, searching for ways to preserve sexual function. They disagreed with their family, who emphasized curing the cancer alone. Japanese men generally hesitate to reveal their sexual problems to their physicians [18, 19]. Moreover, depending on the circumstance, the information provided by each hospital may be biased. Furthermore, there is no educational or consulting system that involves nurses in patients’ sexuality in Japan. Patient involvement in discussions on sexual dysfunction depends on the ability of the individual nurse [20]. Therefore, men and their families may not obtain accurate information concerning treatment, side effects, and sharing emotions, which could cause psychological conflicts. Particularly, healthcare professionals should be empathetic to encourage men to express their sexual problems and needs. Physicians should provide unbiased information and guidance to fulfill those needs. Nurses should aid and support men and their families by having constructive discussions when deciding treatment strategies for prostate cancer and sexual dysfunction. There is a need to provide education for healthcare professionals who may be involved in patients’ sexuality.

One of our participants shared his experience of giving up sexual function to cure cancer by replacing sexual acts with communication and skinship and reported his emotions concerning the loss of sexual life. Generally, prior to treatment initiation, men who face sexual disabilities worry about the foreseeable sexual dysfunction after prostatectomy. However, anticipatory grief strengthens the ability to withstand shocks, prepares the mind to accept the loss, and reduces shock when the failure occurs [21]. This participant had probably predicted that sexual dysfunction would affect his intimate relationship with his wife and prepared his mindset accordingly.

There was no disagreement about ADT use between men and their families. Moreover, only men who underwent ADT shared their grief about losing sexual ability and sex life. Many older adults have no choice but to undergo ADT, which hinders sexual function. Therefore, it is preferable to seek support to handle anticipated grief even before treatment initiation.

### Loss of values related to sexual dysfunction

Every man is afraid of losing his value due to the development of sexual dysfunction. Cancer treatment is very stressful and traumatic. One of the risk factors for cancer-related PTSD is “treatment that affects healthy tissue” [22]. Selvi et al. [23] found that relatively organ-sparing subcapsular orchiectomy was associated with less testicular pain and a lower incidence of PTSD than total orchiectomy. Although cancer-related PTSD assessments require caution, it is important to improve treatments that may eliminate risk factors for PTSD. Although most Japanese surgeons recognize the concerns regarding sexual problems in patients with breast cancer, they do not acknowledge their responsibility in addressing these issues [24]. Physicians presumably have a similar thought process regarding sexual dysfunction in patients with prostate cancer. Japan should strengthen its medical system, including the development of trained teams to provide necessary support to patients with cancer [25]. Physicians should treat prostate cancer and sexual dysfunction with the aim to care for sexual dysfunction in patients with prostate cancer. Nurses should promote a metaphysical view of sexual identity and maintain close contact with patients. Physicians and nurses should collaborate with reproductive experts. They should be required to build a team with sexual care specialists who can mediate and provide consultations on challenging cases.

### Uncertainty concerning the consequences of sexual dysfunction

After treatment, men hoped for recovery but experienced disappointment. In the process, they expected to regain sexual power but realized that it would not happen. Regardless of treatment selection, there was no guarantee of recovery. In this study, those in the radiation therapy group confessed to such experiences. Erectile dysfunction after radiation therapy is not an immediate side effect. The assessment of long-term erectile function is uncertain because of age-related decline [26, 27]. The uncertainty can be distressing, although this ambiguity can be relied on to help men find and maintain hope [28]. Moreover, in the agonizing process, a person changes his view of life, reassesses what is essential after all, focuses on various options, and gains new power [28]. The experience of cancer and treatment-related uncertainty is associated with the cultural background of the man and his family [29]. We advocate personal re-support based on an understanding of the individuals’ beliefs and values.

### Sense of calm with less adverse effects of sexual dysfunction

Many men had sexual dysfunction. However, some of them showed a lower impact of dysfunction. Their remarks focused on changes in their sexual desires and enjoyment, compared with their experience before the treatment, because of aging and decreased sexual life due to their partners’ dyspareunia and death. Others confessed to relationships without sex and their partners’ understanding and acceptance of their agony regarding sexual dysfunction. A patient who underwent ADT stated that he disliked his sexual orientation, adding that the treatment effectively helped him suppress sexual desire. The Japanese government developed regulations concerning sexuality from the late 1870s to the early 1940s, and most Japanese individuals monitored themselves [30, 31]. Comprehensive sex education, which was first established in the Western countries, was initiated in Japan in the late 1970s. However, it has barely permeated the society. In March 2021, the Japanese judicial system ruled that failure to recognize same-sex marriage violated the constitution that stipulated “equality under the law.” At last, homosexuality has been recognized. Indeed, homosexuals faced prejudice for a long time. Many Japanese individuals appear to have denied their sexual orientation. The participants’ age, acceptance by their partners, and their sexual orientation may significantly impact the recognition of sexual dysfunction. We should clarify the factors influencing the cognition of sexual dysfunction and establish a system to assess the need for care.

### Effort to accept sexual dysfunction and management of changed body

Men strived to accept the reality, enjoying daily life without appearing anxious concerning their sexual disabilities and reckoning life values. They searched for ways to control their sexual emotions or orgasms without relying on specialists. Meanwhile, they showed little motivation to seek professional support to resolve their problems, although they desperately attempted to deal with sexual disabilities. In Japan, a public medical insurance system enables people to receive medical treatment for fixed fees. However, few erectile aids that are approved as medical devices are introduced to patients [32]. Intracavernosal injections, which are commonly used for erectile dysfunction treatment in most countries, are not covered by the insurance system in Japan, and to the best of our knowledge, a limited number of hospitals perform protease insertion prostatectomy. If men are diagnosed with workable sexual function and a low risk of extracapsular extension before treatment, they may choose to save the neurovascular bundle of nerves [33] and use phosphodiesterase inhibitors to recover their erectile function [34]. However, a man who underwent prostatectomy and had purchased and used an erection drug online stated the following: “I was terrified of dying with my heart pounding furiously with the drug.” Moreover, if a non-erectile state becomes chronic, the penile corpus cavernosum would become fibrotic. Recovery from this state is difficult, but a healthcare professional can help maintain a man’s erectile function. We hope that experts would offer support for a quick transition to safe treatment for erectile dysfunction.

Our study had some limitations. First, we included a small number of men who received brachytherapy (*n* = 5). Nevertheless, this study is the first to clarify the experience of sexual dysfunction associated with prostate cancer treatment among Japanese men who are reluctant to disclose their sexual troubles. Based on our findings, we intend to prepare a questionnaire to evaluate the psychological and social aspects of sexual dysfunction and combine the questionnaire with an existing scale to assess sexual function. Moreover, we intend to investigate and analyze the kind of care Japanese men with prostate cancer desire for sexual dysfunction. We also intend to develop a system to support those with sexual disorders resulting from prostate cancer treatment; we hope this system would be acceptable by Japanese men and would improve their quality of life.

## Conclusion

We examined the experience of Japanese men with sexual dysfunction associated with various prostate cancer treatments through interviews. The experiences of Japanese men with sexual dysfunction associated with prostate cancer treatments were the following: desire to maintain sexual function and conflict in decision-making concerning the initial treatment for prostate cancer; loss of values related to sexual dysfunction; uncertainty regarding the consequences of sexual dysfunction; sense of calm with less adverse effects of sexual dysfunction at the early treatment stage; effort to accept sexual dysfunction; and management of their changed body at the later treatment stages. Our findings would help the development of appropriate treatment strategies for sexual dysfunction and would help such patients improve their quality of life. Additionally, we hope that our study would stimulate other researchers to perform quantitative studies, which would further highlight experiences related to sexual dysfunction.
